# Evaluation of a Community-Led Program for Primordial and Primary Prevention of Rheumatic Fever in Remote Northern Australia

**DOI:** 10.3390/ijerph191610215

**Published:** 2022-08-17

**Authors:** Anna P. Ralph, Angela Kelly, Anne-Marie Lee, Valerina L. Mungatopi, Segora R. Babui, Nanda Kaji Budhathoki, Vicki Wade, Jessica L. de Dassel, Rosemary Wyber

**Affiliations:** 1 Menzies School of Health Research, Charles Darwin University, Darwin 0810, Australia; 2 Division of Medicine, Royal Darwin Hospital, Darwin 0810, Australia; 3 Rheumatic Heart Disease Australia, Menzies School of Health Research, Darwin 0810, Australia; 4 Sunrise Health Corporation, Katherine 0850, Australia; 5 Northern Territory Government Department of Health, Darwin 0810, Australia; 6 Telethon Kids Institute, Perth 6000, Australia; 7 George Institute for Global Health, Sydney 2000, Australia; 8 Australian National University, Canberra 2610, Australia

**Keywords:** rheumatic fever, rheumatic heart disease, streptococcus, primordial, Aboriginal, environmental health

## Abstract

Environmental factors including household crowding and inadequate washing facilities underpin recurrent streptococcal infections in childhood that cause acute rheumatic fever (ARF) and subsequent rheumatic heart disease (RHD). No community-based ‘primordial’-level interventions to reduce streptococcal infection and ARF rates have been reported from Australia previously. We conducted a study at three Australian Aboriginal communities aiming to reduce infections including skin sores and sore throats, usually caused by Group A Streptococci, and ARF. Data were collected for primary care diagnoses consistent with likely or potential streptococcal infection, relating to ARF or RHD or related to environmental living conditions. Rates of these diagnoses during a one-year Baseline Phase were compared with a three-year Activity Phase. Participants were children or adults receiving penicillin prophylaxis for ARF. Aboriginal community members were trained and employed to share knowledge about ARF prevention, support reporting and repairs of faulty health-hardware including showers and provide healthcare navigation for families focusing on skin sores, sore throat and ARF. We hypothesized that infection-related diagnoses would increase through greater recognition, then decrease. We enrolled 29 participants and their families. Overall infection-related diagnosis rates increased from Baseline (mean rate per-person-year 1.69 [95% CI 1.10–2.28]) to Year One (2.12 [95% CI 1.17–3.07]) then decreased (Year Three: 0.72 [95% CI 0.29–1.15]) but this was not statistically significant (*p* = 0.064). Annual numbers of first-known ARF decreased, but numbers were small: there were six cases of first-known ARF during Baseline, then five, 1, 0 over the next three years respectively. There was a relationship between household occupancy and numbers (*p* = 0.018), but not rates (*p* = 0.447) of infections. This first Australian ARF primordial prevention study provides a feasible model with encouraging findings.

## 1. Introduction

Australia has committed to eliminate rheumatic heart disease (RHD) by 2031 [1]. Indigenous Aboriginal and Torres Strait Islander peoples bear a disproportionate burden of RHD, particularly in remote communities in Northern Australia [2]. Australia has invested in programs to prevent progression of established RHD, but different strategies are needed to prevent new cases. Group A Streptococcus (Strep A) infections precipitate acute rheumatic fever (ARF) in some individuals (usually children, peaking in the 5–14 years age group); severe or recurrent ARF can lead to RHD (usually in young adults), which can lead to heart failure and premature death. Addressing the environmental and structural drivers of these steps in the pathogenesis of ARF and RHD is generally termed ‘primordial’ prevention [3]. Antibiotic treatment of Strep A infections to prevent the abnormal immune reaction of ARF is considered ‘primary’ prevention and can reduce risk by up to 80% [4]. In the NT, 52% of people with an initial ARF diagnosis progress to RHD within 10 years [5]. Improving primordial and primary prevention of ARF in Australia are agreed priorities [6,7]. The large decline in ARF incidence in higher income countries during the early 20th century has been attributed chiefly to primordial prevention [8]. However, no intervention studies have been attempted and there are no models available of how this can be achieved in practice.

Delivery of secondary prevention improved in the Northern Territory (NT) between 2000 and around 2014 [9], and there has been a small decrease in the ARF recurrence rate for those prescribed intramuscular penicillin as secondary prophylaxis [10]. However, the COVID-19 pandemic has set progress back (NT RHD Control Program data, unpublished). Preventative health care can be de-prioritized when health systems are under strain, emphasizing the need for sustainable action to address household crowding and socioeconomic factors, the leading determinants of ARF risk [11,12].

Australian Aboriginal children living in remote communities have the highest documented global burden of impetigo (chiefly attributable to group A Streptococcus [13]) with a median prevalence of 44.5% in children < 15 years [14]. This is likely to be a key driver of high ARF rates in this setting [15,16]. In the absence of a vaccine, practicable primordial prevention comprises access to washing facilities and education about hygiene strategies [17]. Other aspects of primordial prevention—reducing bed-sharing and household crowding—are challenging or impossible without significant government investment into housing stock and maintenance, and strategies to achieve economic gains in remote communities.

Primary prevention comprises timely antibiotic treatment of streptococcal infections to prevent subsequent ARF, proven to be an effective strategy [18]. This should be combined with other measures to reduce onward transmission such as covering skin sores, good cough etiquette, hand hygiene and avoiding bed-sharing and other close contacts while infectious [17]. 

We developed and implemented a three-year community-based, outreach-to-household project to support primordial and primary prevention of streptococcal infections and acute rheumatic fever (ARF) occurrences. ARF is the precursor to RHD. We drew on the existing evidence base for strategies most likely to work [7,17], and on our research with Aboriginal community members about approaches to ARF knowledge-sharing [19] and the need to strengthen community engagement in ARF prevention [9,20]. The project chiefly engaged people with ARF or RHD and their families, but also delivered broad community and school-based activities. Interim findings were published in a formative evaluation [21] and qualitative enquiry explored how the outreach-to-household model was experienced by study participants [22].

In this paper, we report outcomes during the three-year Activity Phase compared with a Baseline Phase. Our aim was to determine whether this model of ARF primordial and primary prevention reduced streptococcal infections and ARF occurrences.

## 2. Materials and Methods

### 2.1. Design and Setting

This is a pragmatic intervention study with Baseline and Activity phases. The Baseline Period was 1 February 2017–31 January 2018. The study Activity period was 1 February 2018–31 January 2021. 

The process of engagement, selection of community sites and employment and training of Aboriginal Community Workers (ACWs) to deliver the study’s goals are described elsewhere [21]. Three Aboriginal communities in the Northern Territory participated, all ‘very remote’ [23]. Site A (population 460 people) and B (population 300 people) commenced in February 2018 and a third community (Site C, population 100 people, a satellite community of Site B) then opted to join, commencing October 2018. Five ACWs were employed for varying periods; Sites B and C were managed by the same ACW. Each site is serviced by one clinic in an accessible location within approximately 2 km of all homes in the community, where care is provided free of charge. Two were under Aboriginal Community Controlled governance and one was under NT Government governance. 

**Figure 1:**
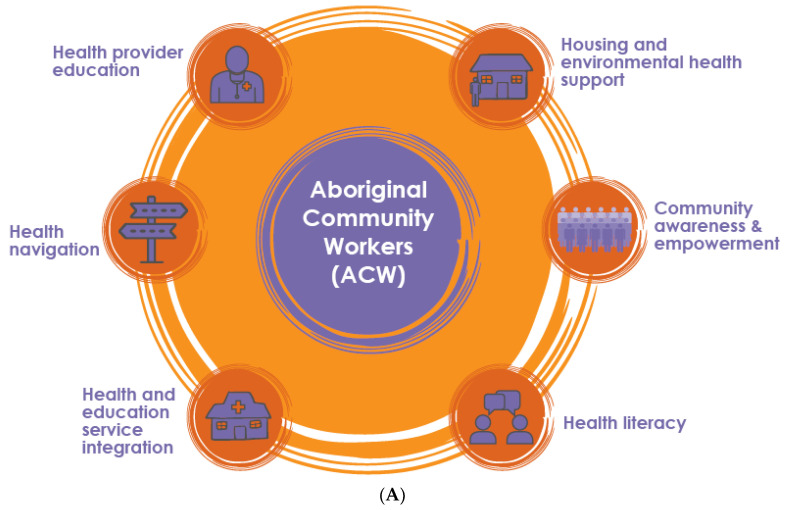

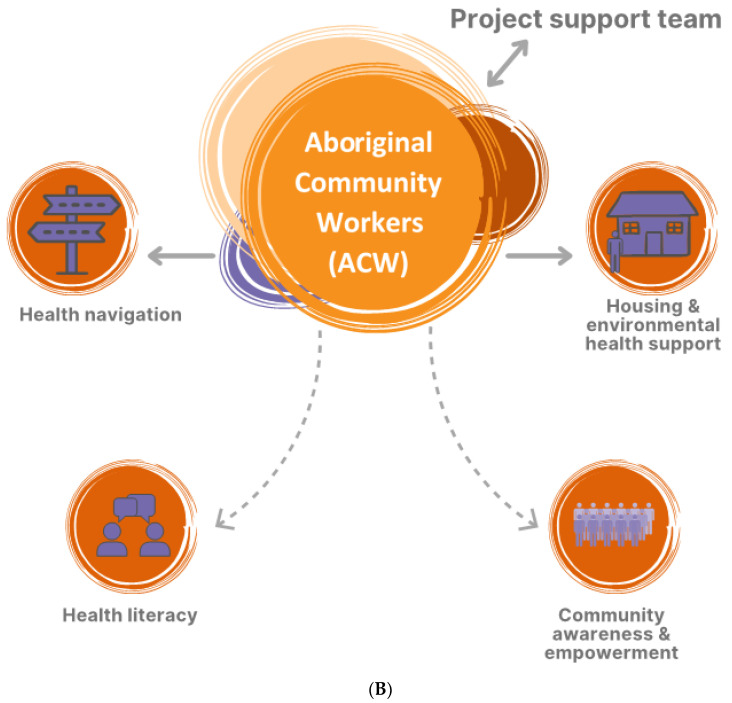
(**A**) Activity domains of the outreach-based support model as originally conceived; (**B**) Actual activity domains best addressed during study implementation (solid arrows: major activities; dotted arrows: minor activities).

The original conceptual model [7] and the actual final study model are shown in Figure 1. In summary, domains of activity aiming to reduce rates of Strep A infection and ARF were: housing and environmental health support; community ARF/RHD awareness and empowerment; health literacy; health education and service integration; health navigation (i.e., assisting clients to access health services); and health provider education. 

### 2.2. Inclusion Criteria 

Households were identified as being eligible by clinic staff and ACWs if a household resident had been diagnosed with ARF or RHD and was currently prescribed secondary prophylaxis. Aboriginal participants of any age who provided consent were eligible. There were no exclusion criteria. Participants could have been diagnosed with ARF or RHD prior to study commencement, or at any stage during the study. If a new diagnosis occurred during the study, the person was referred by clinic staff to the study team, who then invited the person and their family to participate. Participants living with ARF or RHD were considered primary participants and household members were contact participants. Residents of the participating communities receiving a new ARF or RHD diagnosis and requiring secondary prophylaxis could join any time during the study. 

### 2.3. Consent

Written, informed consent was sought by the ACW or members of the study team working with the ACW, from primary and contact participants, in languages appropriate to the community (one Aboriginal language was spoken at Site A and another spoken at Sites B and C). Guardians provided consent if the participant was aged <18 years. Eligible household members were approached at a neutral location such as a meeting place outside the community clinic or shop to discuss the participant information materials and provide consent.

### 2.4. Outcome Measures

The primary outcome measure was potential streptococcal infections in the Baseline and Activity Phases of the study. Secondary outcome measures comprised community-wide ARF occurrences and adherence to secondary prophylaxis. Potential streptococcal infections were defined clinically, as any clinic presentations comprising sore throat (pharyngitis, tonsillitis) or infected skin sore (impetigo) without abscess (abscesses were considered more likely to be attributable to Staphylococcus aureus) [24]. Microbiological results were not obtained since swab collection is neither recommended [25] nor readily available in remote settings. The majority of skin sores in remote NT Aboriginal communities are caused by Group A Streptococcus with clearance of Group A Streptococcus shown to be the only independent predictor of treatment success highlighting the streptococcal etiopathogenesis even if Staphylococci are co-isolated [13]. In international studies, an estimated 20–40% of sore throats may be attributable to Group A Streptococcus [26,27]; therefore, while most sore throats have a viral etiology, we included sore throat as indicative of potential streptococcal infection due to the important historical association between pharyngitis and ARF, and because sore throat is widely used as a proxy for streptococcal infection in high ARF burden settings. 

### 2.5. Data Collection

Household surveys were conducted by ACWs to obtain data on household occupancy, bed-sharing, presence and functionality of health hardware, whether anyone in the house had a potential streptococcal infection since the last survey and if so, what action had been taken (Supplementary Table S1). We hypothesized that self-report might reveal more infections than were seen at the clinic. The original intention was to conduct inspections of health hardware, but this was not considered culturally acceptable, even if inspections were by a trusted community member, so surveys were completed based on householder report instead. During the study it became evident that a range of non-streptococcal skin pathologies such as tinea corporis (*Trichoyphyton rubrum* infection) were being included in these reports. Therefore, a chart illustrating different skin pathologies (impetigo, scabies, scabies with superimposed bacterial infection, tinea) was created (Supplementary Figure S1) for the ACWs to refer to and share with participating families to guide data collection and as a basis for skin health education, focusing on pathologies likely to be streptococcal in etiology. Household data were collected using paper forms and entered into a REDCap (Research Electronic Data Capture) database hosted at Menzies School of Health Research [28]. Household occupancy data collection was facilitated at the outset of the study using a magnetic board (Supplementary Figure S2) [29].

Primary Healthcare Clinic data were manually extracted from clinical records for the Baseline and Activity Phases for primary and contact participants from Baseline period start (1 February 2017) until study end (31 January 2021). We were unable to account for movements into and out of participating communities and applied the assumption that people were resident in the community throughout the study. However, if primary participants withdrew, left the community or died, their data were censored at that date. Clinic data comprised presentations consistent with potential Strep A infection (impetigo, pharyngitis), presentations relating to Strep A sequelae (ARF (including presentations with joint pain suspicious for ARF without a clear diagnosis), RHD, acute post-streptococcal glomerulonephritis (APSGN)), and infectious conditions potentially related to environmental living conditions (scabies, ear infections, boils, upper and lower respiratory tract infections, fever) (Supplementary Table S2) [21]. 

The NT RHD Register, administered by the NT Government Department of Health, is a database of all people known to have ARF and RHD in the NT, which tracks receipt of secondary prophylaxis and collates data on diagnosis and management. Data on ARF occurrences, new RHD notifications, disease priority as recorded in the RHD Register (Priority 1: severe RHD; Priority 2: moderate RHD; Priority 3: mild or no RHD; Priority 4: secondary prophylaxis no longer required) [17] and the delivery of secondary prophylaxis injections received by primary participants in the participating communities, were obtained from Northern Territory RHD Register for the Baseline and Activity Periods. RHD Register data were included from the start of the Baseline period, or from first ARF or RHD diagnosis if that occurred later. 

### 2.6. Data Reporting and Analyses

The nature and frequency of activities implemented by ACWs and the project team were captured in project officer reports, study newsletters and interviews, and are reported elsewhere [21,22].

Household occupancy was expressed as median number of people who slept in the house the night before. Potential streptococcal infections were expressed as numbers and as rates per person-years, where person-years were calculated from start of Baseline until end of Activity Phase Year 3, or censored at the time of departure from the study. Rates were found to be zero-inflated and not normally distributed and a suitable transformation could not be identified. Therefore, the mean was used as a summary statistic, but non-parametric comparator tests were used. Change in rate was calculated using the Kruskall-Wallis test. Change in number of infections was not compared, since participants stayed in the study phases for different periods of time. 

Pearson’s correlation coefficient was used to assess the strength of the relationship between self-reported and clinic-reported infections, and the relationship between household occupancy and self-reported streptococcal infections. 

Adherence to secondary prophylaxis with intramuscular benzathine benzylpenicillin (BPG) every 28 days was calculated from study commencement or the date at which penicillin for ARF prophylaxis was commenced if a new diagnosis occurred during the study. Adherence calculations stopped at the end of the study, or the date at which secondary prophylaxis was ceased by a health care provider or if the patient withdrew, left the study site or died. Percent adherence and ‘days-at-risk’ were calculated for whole communities in the Baseline and Activity Phases [30]. That is, if a person joined in Activity Year 2, their data were included even though they did not have a comparative Baseline Phase. Percent adherence (proportion of scheduled doses received) was considered adequate if ≥80% of scheduled doses were received (i.e., ≥11 doses out of 13 in a 12 month period) [30]. For <12-month periods, an appropriate denominator was used. Extra doses (e.g., more than one dose during a 28-day period) were retained in adherence calculations, but percent adherence was capped at 100%. ‘Days-at-risk’ (DAR) is an adherence measure that accounts for the periods between BPG doses. For DAR calculation, the date the BPG dose was administered is considered day 1 and DAR commence on day 29 if another dose had not yet been administered [30]. DAR were presented as the annual sum of DAR. 

## 3. Results

**Figure 2:**
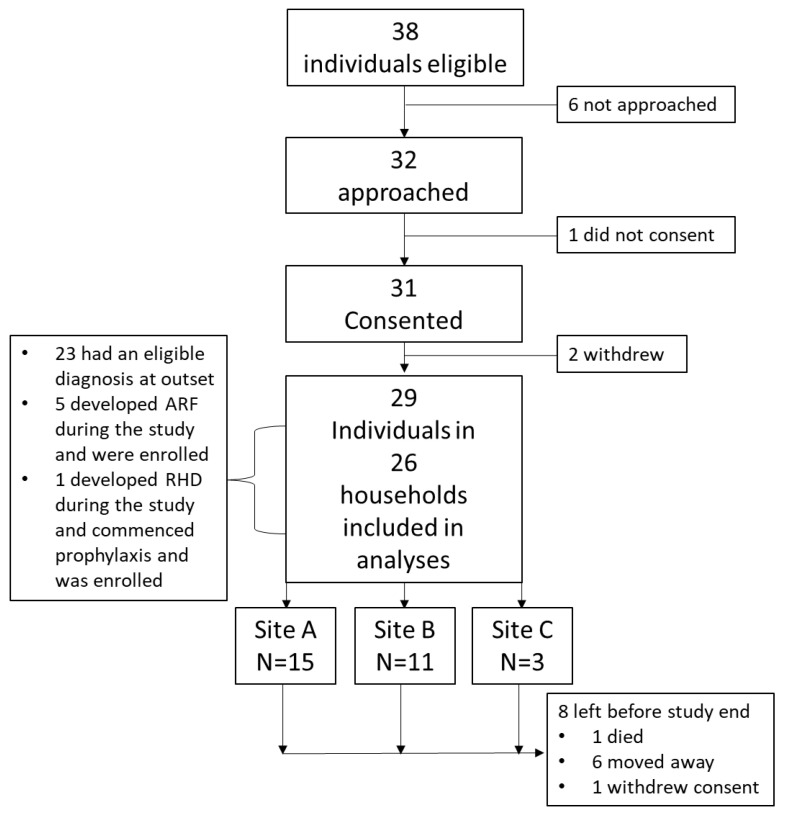
Study enrolment diagram.

Thirty-one primary participants living in 26 households consented out of 38 eligible and 32 approached (Figure 2). Ten left before the end of the study: one died, two withdrew before any data collection commenced, one withdrew during the study, and six moved out of the study community. Those who withdrew did so passively (stopped interacting with the ACW). Sixteen were female and median age at enrolment was 14 (range 7 to 76 years) Table 1. Twenty-three primary participants had a pre-existing diagnosis of ARF and/or RHD with the diagnosis made a median of 5 years before commencement of the Activity Phase (range 3 months to 20 years). During the Activity Phase, a further six were diagnosed with ARF for the first time and one was diagnosed with previously unrecognized RHD; all were commenced on secondary prophylaxis and consented to participate. Entry into and exit from the study and other sentinel events are depicted in Supplementary Figure S4. Twenty-six contact participants living in the same households also consented (between 0 and 7 per household). Total person-years contributed to the study was 196.8 (primary participants: 92.8 person-years; contact participants: 104 person-years).

### 3.1. Implementation of Activities to Address ARF

We used the TIDieR checklist (‘Template for Intervention Description and Replication’) to describe components of the intervention (Supplementary Table S3) [31,32,33,34,35]. Activities undertaken with the aim of reducing Strep A infection and ARF cases are reported in detail elsewhere [21,22] and key learnings, challenges and responses are provided in Supplementary Table S4. As determined through qualitative enquiry [22], the most successful ACW activities were those relating to housing and environmental health support and health navigation. Moderate gains were made in health literacy and community awareness, and limited gains in health provider education and integration with educational systems (Supplementary Table S3) [22]. 

Each household survey occasion provided an opportunity for education about ARF prevention. Some knowledge gain was evident on assessment in the second year of the Activity Phase [21]. ACWs became recognized in the community as ‘go-to’ people, in addition to clinic staff, to provide knowledge and support about ARF. From 2020 (Year Three of the Activity Phase), the ACWs pivoted to additionally providing COVID education (which shares prevention messages with ARF in relation to hand hygiene, cough etiquette and physical distancing) including participation in developing video resources in local languages [36]. ACWs also became RHD ‘Champions4Change’, an avenue for further knowledge gain and advocacy relating to ARF and RHD [37]. 

### 3.2. Clinic-Documented Infections

**Figure 3:**
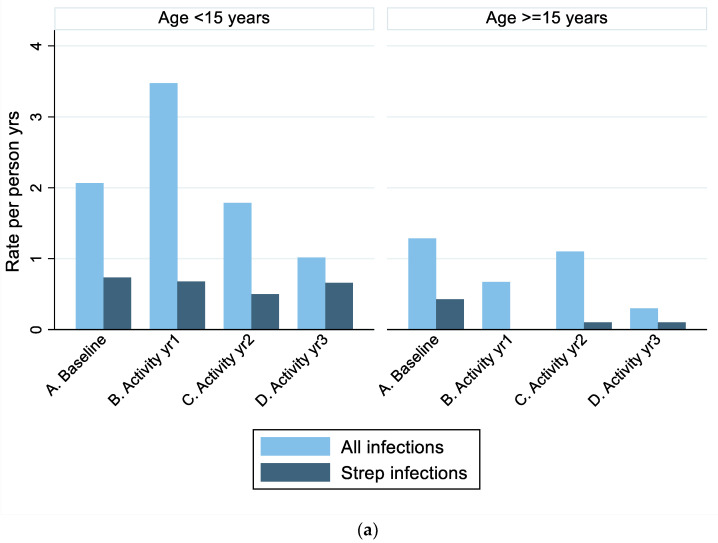

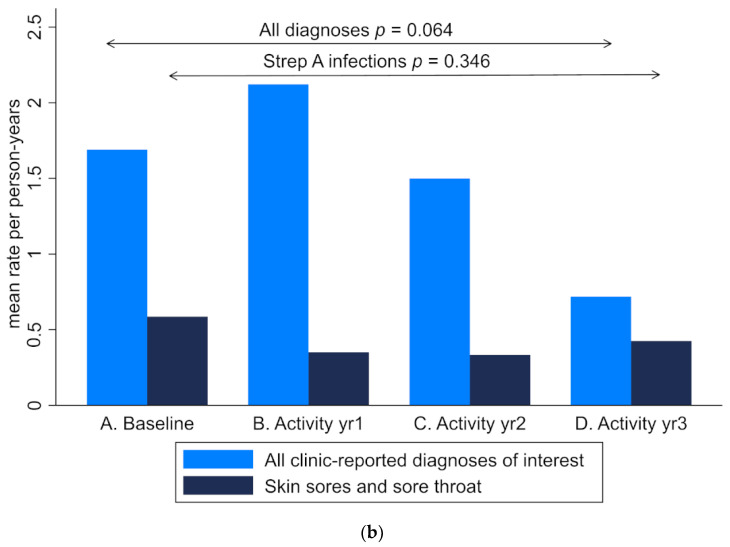
Rates of streptococcal infections in primary study participants. (**a**) By age group; (**b**) All age groups.

Clinic records were obtained for all 55 participants living in 26 households: 29 primary and 26 contact participants. During the whole study, 271 diagnoses of interest were recorded (Table 2), 112 potentially related to Strep A (sore throat, skin sore, ARF, presentation suspicious for ARF, RHD, APSGN). Among primary participants, children aged <15 years had higher rates of all diagnoses (mean rate per person-year: 2.11, 95% CI 1.53–2.69) compared with people aged ≥15 (0.86, 95% CI 0.57–1.15, Kruskall-Wallis *p* = 0.0017) and higher rates of Strep A infections (mean rate per person-year: 0.64, 95% CI 0.43–0.86) compared with people aged ≥15 (0.17, 95% CI 0.03–0.30, Kruskall-Wallis *p* < 0.001) (Figure 3A).

Primary participants had more clinic presentations with relevant diagnoses than contact participants even when presentations relating to ARF or RHD were excluded (Fisher exact, *p* = 0.043). However, primary participants were much younger than contacts (median age 14 years (IQR 9–25) versus 36 years (IQR 29–49), *p* < 0.001). Given the strong association between Strep A risk and age in this study (Figure 3A) and elsewhere [38], we did not include the data from household contacts in conjunction with primary participants and focused the infection data reporting on primary participants only. 

Among primary participants, all relevant clinic diagnoses (including potential Strep A-related conditions) occurred at a mean rate per person-year of 1.69 (95% CI 1.10–2.28) in the Baseline Phase; this increased in Activity Phase Year One to 2.12 (95% CI 1.17–3.07) and fell thereafter (Activity Phase Year Two: 1.50, 95% CI 0.75–2.25; Activity Phase Year Three: 0.72, 95% CI 0.29–1.15) (Kruskall-Wallis *p* = 0.064) (Table 3, Figure 3B). There was no statistically significant change in the rate of clinic-diagnosed potential Strep A infections (*p* = 0.345). (Table 3, Figure 3B). 

Skin sores, which (except one) all occurred in children < 15 years, appeared more common in the Baseline Phase but numbers were small (Table 3). 

### 3.3. Self-Reported Infections

Surveys (*n* = 1304) of the 26 participating households were conducted by ACWs, supported by the project team. New potential streptococcal infections were reported among household members in 36 separate surveys, but only eight indicated that the affected family member went to the clinic (mostly within 1 day of onset) and six indicated they had received a form of treatment. There was no association between household and clinic-reported data. Even when a household survey was conducted on the same day, or within several days of, a clinic presentation, there was no overlap between reports of potential streptococcal infections between the two data sources (r = −0.0056; Supplementary Figure S3). 

### 3.4. ARF Diagnoses

Data from the participating communities (which included two ARF diagnoses during the study period among people who were not enrolled) showed that new cases of ARF decreased during the study period. There were six cases of first-diagnosed ARF during Baseline, five in Activity Phase Year One, one in Activity Phase Year Two and zero in Activity Phase Year Three (Table 4, Supplementary Figure S4). However, recurrent ARF episodes continued to occur during the study period among enrolled participants already prescribed penicillin: zero in Baseline, two in Activity Phase Year One, zero in Activity Phase Year Two and three in Activity Phase Year Three. These five recurrences occurred in four individuals with only one being a definite diagnosis, the others being uncertain (not fulfilling ARF diagnostic criteria) [17]. In addition, a previously undetected case of RHD was diagnosed in Activity Phase Year One (Supplementary Figure S4).

All who experienced recurrent ARF had documented ‘days-at-risk’ due to late penicillin dosing, median 23 days at risk, though one had only 10 days at risk (Supplementary Table S6). In one individual with two ‘ARF recurrences’, specialist pediatricians agreed that the diagnosis was uncertain and ceased the penicillin prescription but ultimately re-instated it, deciding that ongoing secondary prophylaxis was the safer option even though the diagnosis was uncertain. 

### 3.5. RHD Severity

RHD status of the primary participants worsened in one instance (from mild or no RHD to moderate-severity RHD), regressed in one (from mild or no RHD to inactive status, that is, no longer requiring secondary prophylaxis) and stayed the same in 27 people. One elderly individual died (of a vascular cause, with RHD a secondary contributor); this patient had severe RHD at enrolment and throughout follow up.

### 3.6. Household Occupancy and Health Hardware

There was a median of five occupants per house (range 1–16 people) (Table 1). Primary participants reported sharing a mattress with one other person on 20% of surveyed occasions and with ≥2 others on 6% of occasions (attributed to high rates of bed-sharing in Site B, Table 1). The shower was not working on 15 occasions reported by seven different households (1% of surveys; Supplementary Figure S5) and no hot water was available for washing on 19 occasions. Soap was reportedly available almost universally (Table 1). Toilets were not working on eight occasions and households reported having no washing machine available in 19 surveys. At Community B, a community laundromat was established during the second year of the Activity Phase and was reportedly well-utilized. 

The ease of actioning repairs differed across sites, depending on relationships between the ACW and the availability of local Department of Territory Families, Housing and Communities. The Darwin-based project manager was required to help facilitate several repairs. 

### 3.7. Association of Potential Streptococcal Infections with Household Occupancy and Health Hardware

Associations were explored between numbers of people living in households and infections in primary participants. 

**Figure 4:**
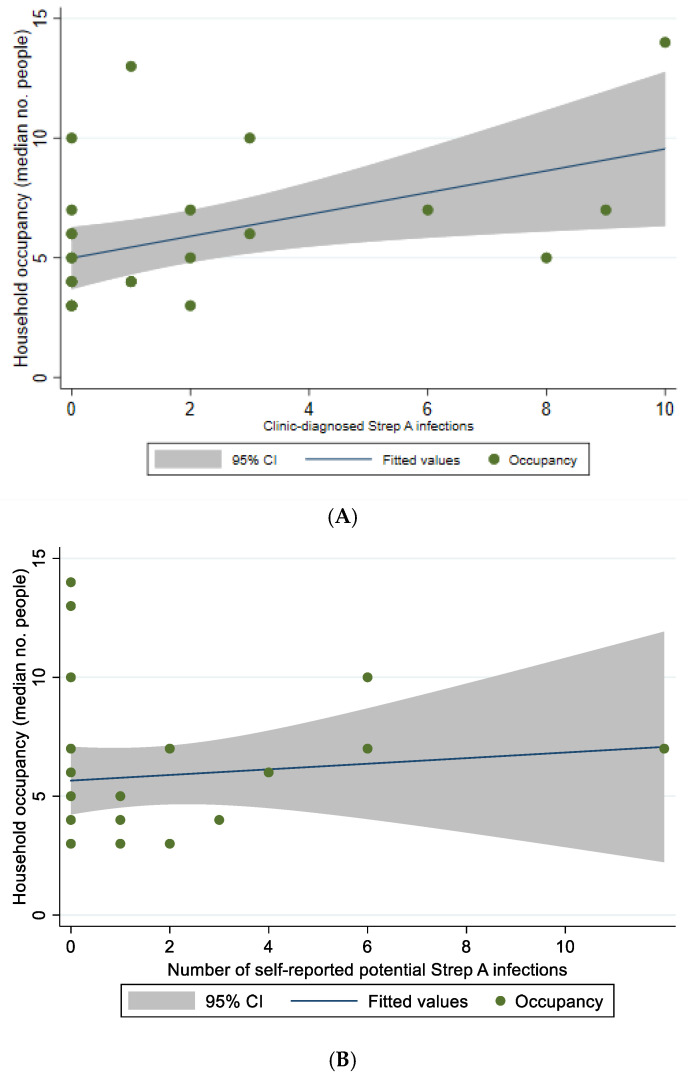
Association between potential Streptococcal infections and median household occupancy. (**A**) Clinic-reported potential Streptococcal Infections (skin sores and sore throats), Correlation coefficient r = 0.46 (*p* = 0.018); (**B**) Self-reported skin sores and sore throats, Correlation coefficient r = 0.10, *p* = 0.595.

There was a statistically significant, directly proportional relationship between numbers of household occupants reported in household surveys and numbers of clinic-reported Strep A infections (correlation coefficient r = 0.46, *p* = 0.018, Figure 4A). However, there was no statistically significant relationship of household occupancy with number of self-reported Strep A infections (correlation coefficient r = 0.10, *p* = 0.595, Figure 4B) or rate of clinic-reported Strep A infections per person years (correlation coefficient r = 0.0747, *p* = 0.4466). 

### 3.8. Delivery of Penicillin Secondary Prophylaxis

The proportion of people receiving ≥80% of scheduled penicillin doses was 77.3% during Baseline compared with 62.5% during the Activity Phase. Number of days at risk per year got worse during the study (median number of days at risk: 24 in Baseline vs. 60 during the Activity Phase, *p* = 0.017, Table 4). 

### 3.9. Association between Penicillin Adherence and Strep A Infections

**Figure 5:**
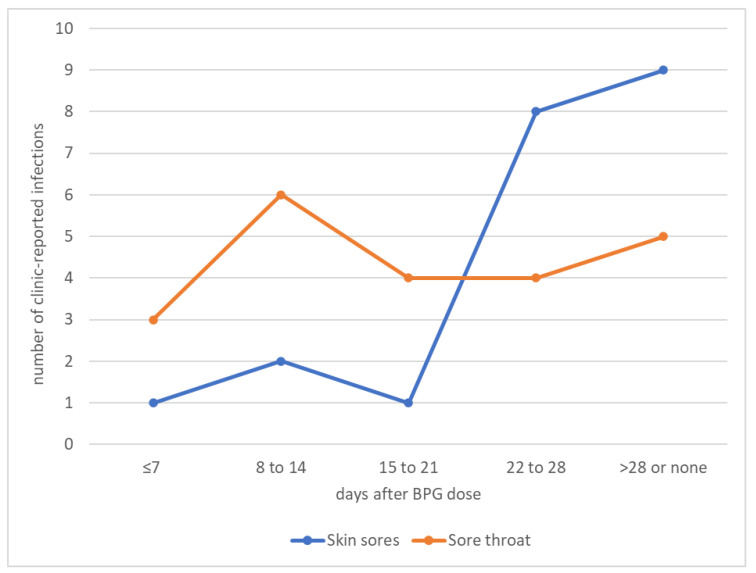
Numbers of potential streptococcal infections by time since last benzathine benzylpenicillin dose.

We examined the relationship between receipt of penicillin by primary participants, and clinic diagnoses of potential streptococcal infections (Figure 5). Recent BPG appeared somewhat protective against incident skin sores. Skin infections (which most reliably represent streptococcal infection) were uncommon until 3 weeks after a BPG dose, at which point numbers increased. Sore throat (less reliably representing streptococcal infection) occurred in similar numbers regardless of timing of prior BPG.

### 3.10. Association between Strep A Infections and ARF

Eleven people were diagnosed with ARF (10) or RHD (1) during the 4 years of this study. Nine of those people had a potential streptococcal infection diagnosed at the clinic prior to their ARF or RHD diagnosis (Supplementary Figure S4), only four of whom were prescribed an antibiotic. One individual had skin sores on two occasions followed by sore throat, followed by ARF. The others had skin sores (6) or sore throat (3) prior to their diagnosis of ARF or RHD. These were not necessarily the triggering streptococcal events given long time intervals in some instances (Supplementary Figure S4) but illustrate the high burden of streptococcal disease in individuals subsequently diagnosed with ARF. 

## 4. Discussion

This study found an initial increase followed by a decrease in clinic presentations for all relevant infections among young people under 15 years during the Activity Phase compared with Baseline (Baseline rate per person-year: 1.69; Activity Phase Year One 2.12; Year Two: 1.50; Year Three: 0.72). This was consistent with our hypothesis that increased awareness of infections might lead to more clinic attendances, followed by a fall in infection occurrences. However, this was not statistically significant (*p* = 0.064), and numbers and rates of the subset of potential Strep A infections and complications (skin sores, sore throats, post-streptococcal sequalae) did not decrease (*p* = 0.346). We saw an encouraging decrease in numbers of new ARF diagnoses during the 4 years of the study from six in one year to none. This is the first substantive study in Australia to report on implementation and outcomes of a community-based study to reduce streptococcal infection rates, and thereby ARF occurrences, through primordial-level interventions.

The potential for Aboriginal community-led interventions to impact on disease rates holds major promise. This study should help inform strategies for scale-up of ARF primordial prevention interventions in Australia. Our approach was well-supported by participating community members, and the addition of Site C at the request of an Aboriginal Community Worker highlights enthusiasm for the project and prioritization of the issue of ARF prevention. Broader roll-out with larger numbers of participants across more communities, coupled with active case finding of streptococcal infections and ARF occurrences, will help answer questions about effectiveness of community prevention activities. 

Uniquely, by linking clinic, register and household survey datasets, we could explore events preceding sentinel clinical diagnoses. We found that BPG appeared to offer protection against skin sores for up to 21 days after a dose. This is consistent with data on waning serum concentrations of BPG after several weeks [39] and highlights the need for penicillin formulations or administration strategies that could provide therapeutic penicillin levels for longer [40]. The lack of evident impact of BPG on sore throat presentations, while acknowledging that very small numbers limited our ability to draw conclusions, could reinforce that skin sores are a better marker of streptococcal burden than sore throats (which are often of viral etiology) at the study sites. We found that ARF recurrences only occurred if BPG dosing was delayed beyond 28 days. Frustratingly for some people, breakthrough recurrences occurred after as few as 10 ‘days-at-risk’. This is consistent with our previous research illustrating protectiveness of BPG against ARF recurrences on the whole [30], but with occasional breakthroughs [41]. Regular BPG still remains the gold standard agent for secondary prophylaxis. The immense burden this imposes on families, communities and health systems is evident when realizing that during this study in three small communities, nearly 1000 BPG doses were delivered. We did not find improvement in secondary prophylaxis adherence. The COVID-19 pandemic had the effect in the NT of deterring people from attending healthcare and causing significant disruption to primary care. We supported the development of health messaging in Aboriginal languages to counter fear and misinformation about COVID and to encourage clinic attendance [42], but we believe COVID-19-related disruptions could have contributed to increases in DAR in the last study year. Research staff were unable to travel to the participating communities for substantial periods of time from 2020 onwards. Alternative explanations for the adherence findings are that newly diagnosed people entering the study may have had different adherence characteristics compared to those in the study during baseline, or that clinic staff may have devolved responsibility of ‘chasing’ patients for penicillin doses to the ACW, who was not primarily responsible for patient recalls. However, observations and qualitative data [22] did not find evidence of that.

A limitation was that six out of 38 eligible participants were not approached to consent, at the discretion of the ACWs. It is possible that non-participants had different characteristics from participants; however, the proportion of eligible people enrolled was high overall (31/38, 82%). We did not capture movements in and out of communities; for rate calculations, we assumed primary participants lived in the community for the whole period or until documented departure. Factors beyond our control may have influenced findings; for example, an Australian Government funded Rheumatic Fever Strategy Primordial Prevention project [43] began operating at one site during our study, but that project remained at only a formative phase when we ceased data collection.

This study highlights missed opportunities for ARF prevention. Not all skin sore and sore throat diagnoses had antibiotics prescribed. While clinical decisions could have been appropriate (e.g., a ‘skin sore’ may have been non-infective), this suggests under-treatment of these conditions. Importantly, findings also highlight the impacts of social determinants of health. Households had high occupancy with surges up to 16 occupants, creating great pressure on washing facilities and opportunity for infection transmission. We found numbers of infections correlated with numbers per household as expected, and as shown previously [44], but rates per person-years did not show a significant relationship. However, given small numbers, we are confident this does not challenge the understood relationship between household crowding and likelihood of acquiring Strep A infection [11].

## 5. Conclusions

Elimination of RHD as a public health problem in less than 10 years, the challenge set by the Australian government target [1], requires urgent action at all levels of prevention and management. Our research provides an approach for bridging policy-to-practice gaps in primordial and primary prevention—evidence which has been lacking, even though improvements in this domain are likely to be the most important way to sustainably reduce ARF burden [7]. While our study had encouraging findings, larger studies incorporating bold environmental health fixes such as more fit-for-purpose housing and highly visible information campaigns, are likely to be needed to achieve better health outcomes through primordial prevention. Primordial prevention is one key piece of the RHD elimination puzzle; this study provides a scalable model to inform socioenvironmental disease control strategies.

## Figures and Tables

**Table 1:** Primary participant demographic, clinical and household characteristics.

	N = 29 Participants (26 Households) *
Sex	
Female	16 (55%)
Male	13 (45%)
Secondary prophylaxis prior to activity phase commencement	
Diagnosed before study commencement (1 February 2018): N (%)	22 (76%)
Duration of secondary prophylaxis in those with prior diagnosis (median, range)	5.2 years (0.2 to 24.2 years)
Diagnosed during the study (1 February 2018 onwards): N (%)	7 (24%)
Median age at enrolment (range)	14 years (7–76)
Site A (15 participants)	13 years (7–50)
Site B (11 participants)	15 years (7–39)
Site C (3 participants)	32 years (14–76)
Disease severity at enrolment: N (%)	
Severe RHD	4 (14%)
Moderate RHD	1 (3%)
History of ARF or RHD requiring secondary prophylaxis	24 (83%)
Inactive disease not requiring secondary prophylaxis *	0
Median number in household (range): N (%)	5 (1–16)
Site A	5 (3–16)
Site B	5 (2–15)
Site C	4 (3–12)
Primary participant sharing a mattress with ≥1 other: Number of surveys (%)	338/1302 (26%)
Site A	17/660 (3%)
Site B	283/456 (62%)
Site C	37/186 (20%)
Primary participant sharing a mattress with ≥2 others: N surveys (%)	78/1302 (6%)
Site A	0/660 (0%)
Site B	78/456 (17%)
Site C	0/186 (0%)
Soap unavailable, all sites: N surveys (%)	3/1305 (0.3%)
Shower not working, all sites: N surveys (%)	15/1304 (1%)
No hot water in shower, all sites: N surveys (%)	19/1303 (1%)
Toilet not working, all sites: N surveys (%)	8/1303 (0.5%)
No washing machine, all sites: N surveys (%)	19/894 (2%)

* The duration of secondary prophylaxis according to national guidelines [17] depends on the certainty of ARF diagnosis (possible, probable, definite), age at diagnosis, RHD severity, presence of cardiac involvement and whether RHD is preceded by a recognized ARF episode.

**Table 2:** Clinic-reported relevant diagnoses during the whole study.

		Primary Participant	Contact Participant	Total
	N	N = 29	N = 26	
Potentially related to Strep A	ARF	14	0	14
	ARF possible	4	0	4
	ARF probable	2	0	2
	ARF definite	8	0	8
	RHD *	1	0	1
	Acute post-streptococcal glomerulonephritis	2	0	2
	Skin sore	21	13	34
	Sore throat	22	18	40
	Joint pain possibly indicative of ARF	4	3	7
Potentially related to environmental health conditions	Scabies	8	7	15
	Skin boil	14	18	32
	Skin/soft tissue infection **	4	0	4
	Ear infection	17	11	28
	Fever	11	6	17
	Fungal skin infection	18	3	21
	Lower respiratory tract infection	13	19	32
	Upper respiratory tract infection	6	4	10
	**TOTAL**	**169**	**102**	**271**

* New diagnosis of RHD without recognized prior ARF; ** Skin/soft tissue infection other than skin sore or boil e.g., cellulitis, fasciitis.

**Table 3:** Clinic data for diagnoses of interest in primary participants.

		Baseline	Activity Yr1	Activity Yr2	Activity Yr3
		All diagnoses			
Counts	All ages	49	59	36	16
	<15 years	28	50	23	13
	≥15 years	21	9	13	3
Rates	All ages	1.69 (1.10–2.28)	2.12 (1.17–3.07)	1.50 (0.75–2.25)	0.72 (0.29–1.15)
	<15 years	2.07 (1.05–3.08)	3.48 (1.96–4.99)	1.78 (0.54–3.02)	1.01 (0.33–1.70)
	≥15 years	1.28 (0.62–1.94)	0.67 (0.11–1.23)	1.10 (0.39–1.81)	0.30 (−0.05–0.64)
		Skin sore			
Counts	All ages	10	5	2	6
	<15 years	10	5	2	5
	≥15 years	0	0	0	1
Rates	All ages	0.34 (0.70–0.62)	0.17 (0.26–0.32)	0.08 (−0.04–0.20)	0.30 (0.06–0.54)
	<15 years	0.67 (0.17–1.16)	0.33 (0.06–0.60)	0.14 (−0.67–0.35)	0.44 (0.05–0.84)
	≥15 years	0	0	0	0.1 (−0.12–0.32)
		Sore throat			
Counts	All ages	7	5	6	4
	<15 years	2	5	5	4
	≥15 years	5	0	1	0
Rates	All ages	0.21 (0.2–0.46)	0.18 (−0.5–0.41)	0.25 (−0.01–0.51)	0.21 (−0.04–0.48)
	<15 years	0.13 (−0.06–0.33)	0.34 (−0.11–0.80)	0.35 (−0.07–0.79)	0.37 (−0.08–0.82)
	≥15 years	0.36 (−0.07–0.79)	0	0.10 (−0.13–0.33)	0

**Table 4:** Adherence to secondary prophylaxis with intramuscular benzathine penicillin G injection.

		Baseline Phase	Activity Phase				
			Years 1–3				*p* Value ^‡^
		1 February 2017–31 January 2018	1 February 2018–31 January 2021	Year 1	Year 2	Year 3 ^†^	
Participants contributing data	Number	22	28	27	23	22	
Benzathine penicillin G doses administered for ARF secondary prophylaxis	Number	233	755	272	256	227	
Proportion of scheduled doses * received	≥80%	17/22 (77%)	45/72 (63%)	16/27 (59%)	16/23 (67%)	13/22 (59%)	0.201
	<80%	5/22 (23%)	27/72 (38%)	11/27 (41%)	7/23 (30%)	9/22 (40%)	
Days at risk	Number of days at risk per year: median (IQR)	24 (9–80)	60 (35–106)	52 (28–94)	66 (45–111)	75 (49–106)	0.017

* Number of scheduled doses per 12 months = 13 (once every 28 days); ^†^ Year 3 impacted by COVID-19 causing health service disruption; ^‡^ Baseline versus whole Activity phase.

## Data Availability

Data are included in the manuscript and Supplementary Materials.

## Supplementary Materials

The following supporting information can be downloaded at: https://www.mdpi.com/article/10.3390/ijerph191610215/s1, Table S1: Sources of data, frequency and mode of collection; Table S2: Diagnoses extracted from clinic records; Table S3: Description of study intervention activities according to the Template for Intervention Description and Replication (TIDieR) checklist; Table S4: Project implementation—key messages, challenges and responses; Table S5: Community-wide ARF and RHD occurrences during the study at Sites A, B and C.; Table S6: Intramuscular benzathine benzylpenicillin dosing prior to recurrent ARF among primary study participants; Figure S1: Skin educational resource for Aboriginal Community; Figure S2: Example of use of magnetic whiteboard to determine household occupancy; Figure S3: Relationship between self-reported and clinic-diagnosed potential streptococcal infections per household; Figure S4: Schematic showing entry and exit from the study of the primary participants, new diagnoses of ARF or RHD, and preceding potential streptococcal infections; Figure S5: Status of health hardware reported from household surveys.
