# Supplementary material for: Evaluation of a Community-Led Program for Primordial and Primary Prevention of Rheumatic Fever in Remote Northern Australia
Source: Int J Environ Res Public Health. 2022 Aug 17;19(16):10215. doi: 10.3390/ijerph191610215 (PMC9407981; doi:10.3390/ijerph191610215)

Supplementary Materials

Table S1: Sources of data, frequency and mode of collection

**Data**	**Source**	**Planned frequency**	**Actual frequency**	**Baseline data available**
Household surveys on<br>- household occupancy and infrastructure<br>- self-reported potential streptococcal infections (impetigo, pharyngitis)	Self-reported by household member	Weekly	Approximately fortnightly (variable interval between surveys)	No
Primary Healthcare Clinic data on diagnoses of relevance including streptococcal infections (impetigo, pharyngitis)	Recorded by clinic, collected by researchers	Continuous	Continuous	Yes
RHD Register data on secondary prophylaxis injection administration	Provided by RHD register	Continuous	Continuous	Yes
RHD register data on ARF occurrences (first diagnoses and recurrences)	Provided by RHD register	Continuous	Continuous	Yes
Interviews and Project officer field notes<br>*These data reported in separate outputs*	ACWs<br>Participants<br>Clinic staff<br>Project officers	Intermittent	Intermittent	No

Figure S1: Skin educational resource for Aboriginal Community Workers

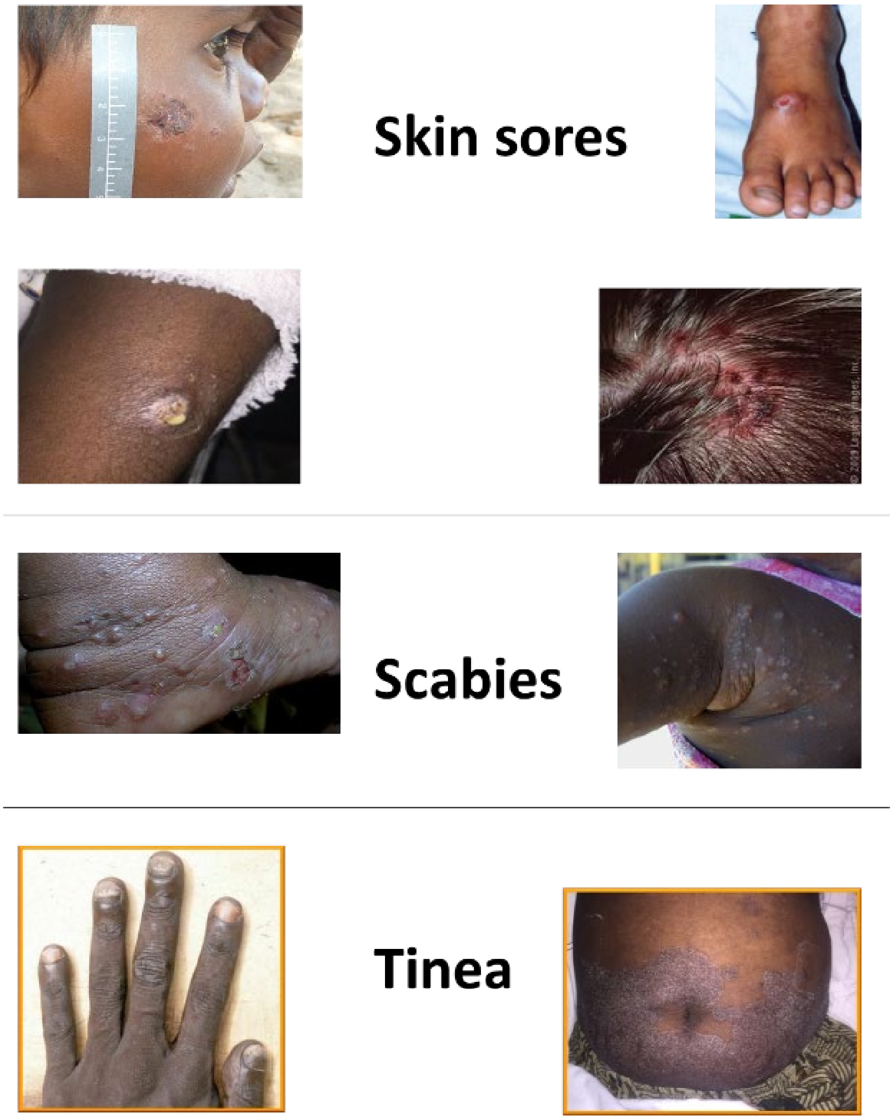

Figure S2: Example of use of magnetic whiteboard to determine household occupancy.

Large magnet = adult; small magnet = child; each square represents one room of the house

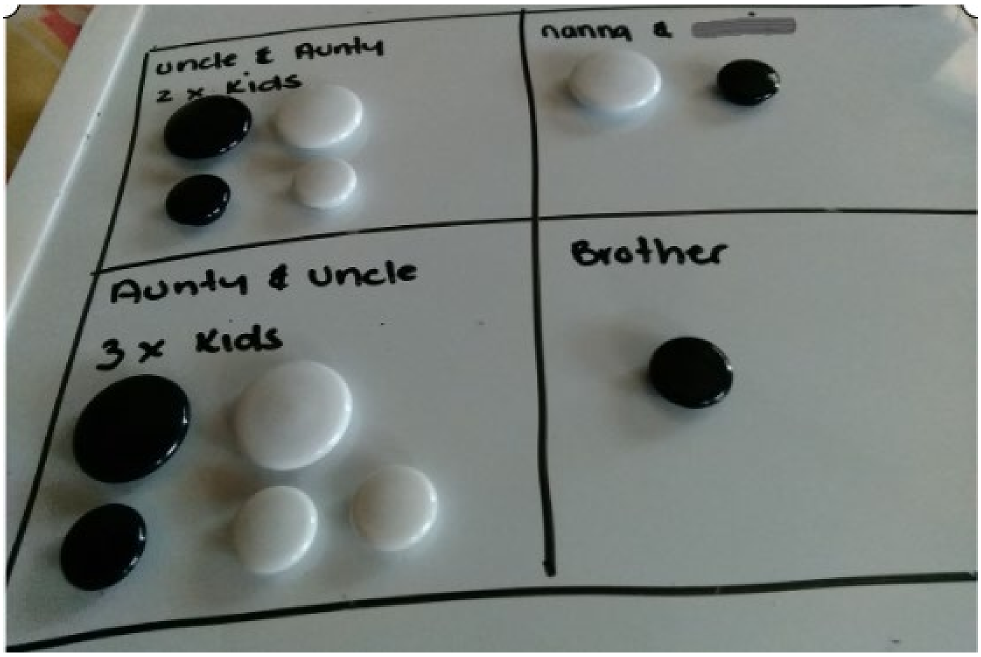

Table S2: Diagnoses extracted from clinic records

	Diagnosis		Diagnosis
1	ABSCESS;SKIN	26	OTITIS MEDIA;SUPPURATIVE;ACUTE
2	BOIL;SKIN	27	OTITIS MEDIA;SUPPURATIVE;CHRON
3	BRONCHIOLITIS;ACUTE	28	PAIN;EAR
4	BRONCHIOLITIS;CHRONIC	29	PAIN;THROAT
5	BRONCHITIS	30	PHARYNGITIS
6	CARBUNCLE	31	PHARYNGITIS;ACUTE
7	CARDITIS	32	PHARYNGITIS;CHRONIC
8	CARDITIS;RHEUMATIC;ACUTE	33	PHARYNGITIS;STREPTOCOCCAL
9	CARDITIS;RHEUMATIC;CHRONIC	34	PNEUMONIA
10	CHEST INFECTION ACUTE	35	PYODERMA
11	FEVER	36	RASH;FUNGAL
12	GLOMERULONEPHRITIS	37	RHEUMATIC FEVER
13	GLOMERULONEPHRITIS;ACUTE	38	RINGWORM
14	GLOMERULONEPHRITIS;CHRONIC	39	RISK FACTOR;RHEUM HEART DISEAS
15	IMPETIGO	40	SCABIES
16	INFECTION;CHEST	41	SCABIES;CRUSTED
17	INFECTION;FUNGUS;SKIN	42	SCABIES;CRUSTED;NOTIFIED
18	INFECTION;LOWER RESP TRACT	43	SORE THROAT
19	INFECTION;THROAT	44	SORE THROAT;STREPTOCOCCAL
20	INFECTION;UPPER RESP TRACT	45	SORE(S);SKIN
21	LOWER RESP TRACT INFECTION	46	TEMPERATURE;HIGH
22	OTITIS MEDIA	47	TINEA CORPORIS
23	OTITIS MEDIA (WITH);EFFUSION	48	ULCER;SKIN
24	OTITIS MEDIA;ACUTE	49	POLYARTHRITIS
25	OTITIS MEDIA;CHRONIC	50	JOINT PAIN

Table S3: Description of study intervention activities according to the Template for Intervention Description and Replication (TIDieR) checklist

Item	Description						
**Name**	'Secondary prophylaxis Plus': the Northern Territory component of the End RHD Communities project						
**Why**	Secondary prophylaxis is a well-established way to reduce adverse outcomes from acute rheumatic fever (ARF); but more is needed since adherence to secondary prophylaxis is not always 100%, and breakthrough episodes can occur. In addition to secondary prophylaxis, primordial and primary prevention needs to be strengthened to stop people who have had ARF from getting it again, and to stop other people at risk from getting it in the first place. This study supported primordial and primary prevention or ARF.						
**When**	February 2017-January 2021						
**Interventio n components**	**Employment of Aboriginal Community Workers**	**Health navigation**	**Housing and environmental health support**	**Health literacy (for ACWs and for community members)**	**Education integration**	**Health provider education**	**Community awareness and empowerment**
**Rationale**	Employing Aboriginal people who live in communities with a high burden of ARF and RHD has been identified as a priority in a number of previous studies [1, 2].	Health or care navigation happens formally or informally when a person is supported to engage with the health service. In New Zealand, Māori Whānau Support Workers have been an important part of the integrated rheumatic fever response to support high risk families [3, 4].	Living in crowded houses is the greatest modifiable risk for Strep A and other childhood infections [5]. Other environmental housing issues, including access to health hygiene infrastructure, are also contribute to the burden of disease. ACW can have a role in documenting housing status and helping to identify pathways towards repairs and tenancy issues.	Awareness and understanding of Strep A infections, ARF and RHD is limited in a number of remote Aboriginal and Torres Strait Islander communities, including in households affected by the disease. This limited health literacy may be a barrier to seeking healthcare [6].	Pre-school and school aged children are at particular risk of skin sores and sore throats [7]. Education through schools can provide an important avenue for children and teachers to learn about the importance of prompt assessment and treatment of these conditions.	Turnover of healthcare providers in remote communities is enormous – half of nurses stay in each clinic only 4 months; 80% have left by 12 months [8]. This poses a barrier to ensuring staff are sufficiently trained in assessment and treatment of skin sores, sore throat, ARF and RHD.	Education supports the denormalization of skin sores throughout the whole community, extending beyond high priority populations (households affected by ARF/RHD and school-aged children) and awareness of health impacts of crowding.
**What**	- Recruitment, retention and training of ACWs to provide information	- ACW encouraging household members with	- Discussion about housing maintenance issues during fortnightly	- Household education during fortnightly visits	- School visits and hygiene education sessions	- One training session for health care staff conducted at	- Informal ACW conversations with community members

	and support to households impacted by Strep A, ARF and RHD.	skin sores and sore throats to clinic for assessment and treatment.<br>- ACW supporting and encouraging people living with ARF/RHD to attend for secondary prophylaxis injections.	household visits and escalation of these issues to relevant authorities as able.			project initiation and ongoing opportunistic education and discussion	- School-based education and engagement
**Materials**	Employment contract	Not applicable	Flip chart; contact number for local housing support officer; emails and meetings with a senior Department of Housing representative	Flip chart; face to face education and discussion	Visits by ACWs and project staff to schools; engaging school children in activities; presentation to teachers / principles	Clinic staff 'in-services' provided by project officer or study investigators	Knowledge sharing in communities led by ACWs
**Who provided**	Menzies School of Health Research	ACWs	ACWs, Project manager, Department of Housing representatives	- Health literacy for ACWs provided by project manager, study investigators, guest educators (microbiologists, doctors) at Menzies School of Health Research and Royal Darwin Hospital<br>- Health literacy for community members provided by ACWs, Project manager	ACWs	Project manager, study investigators	ACWs

**How**	Casual contracts	ACWs engaged with participants informally in the community and supported healthcare attendance by providing advice or escorting people to the clinic	ACWs asked participants if they had any health hardware problems. They reported these to the local housing officer. If action was delayed, the ACW escalated this to the Project manager, who communicated with a senior Department of Housing representative,	- Health literacy for ACWs comprised workshops and roundtable discussions aided by diagrams, anatomical models and flip charts<br>- Health literacy for community members comprised discussions in convenient community locations (outside the local shop, under a tree, at the clinic), aided by diagrams and flip charts	School children were engaged in educational activities such as hand washing activities (‘Glitterbug™’), creating a song about ARF prevention. The project manager and/or study investigators explained the purpose of the project to teachers and principles and provided education on recognition of strep infections and ARF	The project manager and/or study investigators explained the purpose of the project to clinic staff (doctors, nurses, Aboriginal Health Practitioners) and principles and provided education on diagnosis and management of strep infections, ARF and RHD. Hard copied of the ARF/RHD treatment guideline[9] were shared and links to the online version provided.	ACWs upskilled community members in preventative health knowledge through informal discussions and engagement
**Where**	ACWs lived and worked in their communities	In communities (Sites A, B and C)	In communities (Sites A, B and C)	- Health literacy for ACWs: in communities and in Darwin at Menzies and Royal Darwin Hospital<br>-Health literacy for community members: in communities	At community school	At community health clinic	In communities (Sites A, B and C)
**How much**	Continually for 3 years	Ongoing regular task for 3 years	Ongoing regular task for 3 years	Intermittent over 3 years	Intermittent over 3 years	Intermittent over 3 years	Intermittent over 3 years

**Tailoring**	Allowed	Allowed	Allowed	Allowed	Allowed	Allowed	Allowed
**Modifications**	ACWs worked in pairs at Site A and alone at Sites B & C apart from a brief period when two were employed at Site B.	This activity was interpreted somewhat differently depending on the skills, interests and connections of the ACWs	The ease of actioning repairs differed across sites, depending on relationships between the ACW and the local Department of Housing representative and availability of the local Department of Housing representative. The Darwin-based project manager was required to help facilitate several repairs.	Health literacy for ACWs brought the ACWs together at Menzies on occasions to ensure the same training was being received; other training was tailored to needs and interest. The ACW form Site B/C participated in training on how to do an echocardiogram.	This activity was interpreted somewhat differently depending on the skills, interests and connections of the ACWs. Engagement with schools was extensive at Site B, but limited at Sites A and C (overall 'reach' summarised below as 'Good').	Differed at different sites deepening on interest, availability and travel opportunities. Study investigator A.P.R only visited Site A to provide in-services. Project manager delivered in-services at both sites.	No specific activities beyond health literacy and health navigation support took place to promote awareness and empowerment
**How well (reach)***	Good	Very good	Very good	Moderate	Good	Limited	Moderate

*Reach graded as limited, moderate, good, very good, drawing on qualitative data[10] and quantitative data included in this paper and the formative evaluation[11]

References

1. Haynes, E.; Marawili, M.; Marika, B.M.; Mitchell, A.G.; Phillips, J.; Bessarab, D.; Walker, R.; Cook, J.; Ralph, A.P. Community-based participatory action research on rheumatic heart disease in an Australian Aboriginal homeland: Evaluation of the 'On track watch' project. *Eval. Progr. Plan*. **2019**, *74*, 38–53.
2. Read, C.; Mitchell, A.G.; de Dassel, J.L.; Scrine, C.; Hendrickx, D.; Bailie, R.S.; Johnston, V.; Maguire, G.P.; Schultz, R.; Carapetis, J.R.; et al. Qualitative Evaluation of a Complex Intervention to Improve Rheumatic Heart Disease Secondary Prophylaxis. *J. Am. Heart Assoc.* **2018**, *7*, e009376
3. Health Navigator New Zealand. Maori Health—Support. 2021. Available online: https://www.healthnavigator.org.nz/videos/m/m%C4%81ori-health-videos/m%C4%81ori-health-support/ (accessed on 5 June 2022).
4. Anderson, A.; Mills, C.; Eggleton, K. Whanau perceptions and experiences of acute rheumatic fever diagnosis for Maori in Northland, New Zealand. *N. Z. Med. J.* **2017**, *130*, 80–88
5. Coffey, P.M.; Ralph, A.P.; Krause, V.L. The role of social determinants of health in the risk and prevention of group A streptococcal infection, acute rheumatic fever and rheumatic heart disease: A systematic review. *PLoS Negl. Trop. Dis*. **2018**, *12*, e0006577.
6. Mitchell, A.G.; Belton, S.; Johnston, V.; Wopurruwuy, G.; Ralph, A.P. "That heart sickness": young Aboriginal people's understanding of rheumatic fever. *Med. Anthropol.* **2019**, *38*, 1–14. doi:10.1080/01459740.2018.1482549
7. Bowen, A.C.; Mahe, A.; Hay, R.J.; Andrews, R.M., Steer, A.C., Tong, S.Y.; Carapetis, J.R. The Global Epidemiology of Impetigo: A Systematic Review of the Population Prevalence of Impetigo and Pyoderma. *PLoS ONE* **2015**, *10*, e0136789.
8. Wakerman, J.; Humphreys, J.; Bourke, L.; Dunbar, T.; Jones, M.; Carey, T.A.; Guthridge, S.; Russell, D.; Lyle, D.; Zhao, Y.; et al. Assessing the Impact and Cost of Short-Term Health Workforce in Remote Indigenous Communities in Australia: A Mixed Methods Study Protocol. *JMIR Res. Protoc.* **2016**, *5*, e135

9. RHD Australia (ARF/RHD Writing Group). *The 2020 Australian Guideline for Prevention, Diagnosis and Management of Acute Rheumatic Fever and Rheumatic Heart Disease*, 3rd ed.; Menzies School of Health Research: *Darwin*, Australia, 2020. Available online: https://www.rhdaustralia.org.au/arf-rhd-guideline (accessed on 5 June 2022).
10. Kerrigan, V.; Kelly, A.; Lee, A.M.; Mungatopi, V.; Mitchell, A.G.; Wyber, R.; Ralph, A.P. A community-based program to reduce acute rheumatic fever and rheumatic heart disease in northern Australia. *BMC Health Serv. Res.* **2021**, *21*, 1127.
11. Wyber, R.; Kelly, A.; Lee, A.M.; Mungatopi, V.; Kerrigan, V.; Babui, S.; Black, N.; Wade, V.; Fitzgerald, C.; Peiris, D.; et al. Formative evaluation of a community-Anderson Anderson based approach to reduce the incidence of Strep A infections and acute rheumatic fever. *Aust. N. Z. J. Public Health* **2021**, *45*, 449–454.

Table S4: Project implementation - key messages, challenges and responses

**Key points**	Key strengths of the Aboriginal Community Workers were in the priority areas of **health navigation** and **environmental health support**. Future projects should draw on these strengths by building on these activities, whereas other activities shown in the study model may be better undertaken by other experienced project staff.	
	Community ownership and leadership are crucial to devise approaches appropriate to different settings and reaching out effectively to community members to share RHD prevention knowledge and strategies. However, Aboriginal Community Workers requested **stronger support** from the project team including **more intensive training opportunities** and on-the-ground support.	
	While there is an important place for disease specific health interventions in primary, secondary and tertiary prevention and care, comprehensive programs focusing on general healthy lifestyle advice and opportunities to reduce risks of communicable and non-communicable diseases would make more sense, rather than implying that some healthy lifestyle choices such as good nutrition and not smoking are less important due to their lack of proven association with RHD.	
**Key messages from Aboriginal Community Workers[10]**	*"Indigenous people working on the ground from that community, it works well. It doesn't work if an outsider thinks that they can come in...You got to get local people on the ground to do the job. You can work beside that person, we can show you, we can help you. But you always got to remember Indigenous people have the first preference and they are the one that knows..."* – Annie, ACW	*"[I said] 'We don't want [your child] to have that bad valve….He might go to Darwin or Adelaide, get operation, get sick. Show [your son] that.' So she went, 'Okay, [son] come here. [Son] if you don't get that needle, you'll have this.' He looked in there, 'That valve in your heart, bad one and if you get needle all the time you get good valve, it'll be better,' she said to him…..when we showed him that, [he said] 'Okay then Mum, what you waiting for? We got to go now, got to go get that needle now'. - Sylvia, ACW*
**Project domain**	**Findings and Challenges**	**Response and changes implemented**
**ACW employment and experience**	- ACWs face high competing demands on their time due to other paid and unpaid roles, family commitments, and high frequency of tragedies in the community that they must respond to.<br>o As an example, a Darwin-based workshop was not able to be attended by one ACW since their young nephew and sister both died within a few days of the workshop. Then the other ACW's young brother died at around the time of the next Darwin workshop.<br>- Having larger pools of staff at each site can help provide flexibility<br>- ACWs want to work in a team	- Providing flexible employment arrangements is vital<br>- Establishing culturally appropriate staff counselling and condolence responses is high priority<br>- Efforts were made to employ additional ACWs<br>- We encouraged the engagement of ACWs with clinic staff, especially Aboriginal Health Practitioners

	- Allocation of tasks should play to the strengths of the employees<br>- ACWs want hands-on training opportunities e.g. learning to do screening echocardiograms, visiting clinics or hospital wards<br>- ACWs also want the chance to visit each other's communities to see how ARF care is provided elsewhere	- We shifted the focus to health communication and care navigation, not on data collection and other study components<br>- In the last year of the study, one ACW received training in echocardiography and, in collaboration with another study team, helped to initiate and lead a community-based screening exercise in her community which identified new cases of RHD<br>- ACWs visited hospital wards and clinics
**Health navigation**	- ACWs were well placed to support health navigation. This was due to trusting relationships between ACWs and families and the authority attributed to ACWs through the project.	- Use of Menzies-branded T-shirts gives ACWs a sense of authority to take on roles as care navigators and educators<br>- Study team supported positive relationships between ACWs and clinic staff
**Housing and Environmental Health support**	- Plumbing and water problems were common<br>- ACWs were able to effectively work in this space and provide helpful solutions<br>- Where outstanding issues needed to be escalated to the Department of Housing, the project manager was able to do this	- Focusing on sustainability (transfer of responsibility form ACWs or project manager to community members) is the focus for the next stage<br>- Primordial prevention projects must fundamentally seek to improve and increase housing stock
**Health Literacy**	- ACWs becoming ARF/RHD experts, being recognised by community members as the go-to people for information, and sharing knowledge, has been the pivotal activity of the study<br>- However, knowledge among community members of ARF/RHD remained limited throughout involvement in the study.<br>- Culturally appropriate processes for effective knowledge transfer differ among communities and between individuals in a community.<br>- Of most importance is to focus on health literacy of how to prevent ARF, and what to do if someone has had ARF or has RHD.	- Resources have been co-designed between ACWs and project staff that meet local needs. These are mostly pictorial but have some words in Plain English. Neither of the Aboriginal languages used at the study sites are commonly used as written languages, hence the ACWs asked that any necessary writing be in English, which they will translate and embellish in their own languages<br>- Focus on overcoming misconceptions: ARF is not genetic, it is chiefly environmental, and risk is modifiable<br>- Focus less on the complex causative trail of ARF and more on how to stop it

	- Where knowledge gain among family members was apparent, motivation or structural capability to implement behaviour change was lacking in some domains, even though the model was intended to support structural changes through care navigation and housing fixes.<br>o e.g. families learnt about household crowding being a risk factor for strep infection and ARF, and took action to increase bed spacing, but ultimately need more durable solutions i.e. houses with more bedrooms	- Next stages of the project will seek stronger engagement with Department of housing including advocacy for housing solutions, based on personal stories arising from the study participants.
	- ACWs contributed to small improvements in the health literacy of community members and supported awareness and empowerment; but this was limited by disease complexities.<br>- ACWs requested more training to address knowledge gaps and improve knowledge transfer to families.<br>- ACWs indicated that they preferred paper-based resources rather than videos or apps for electronic devices	- More Darwin based workshops bringing together ACWs from the participating sites, and involving more trainers, health practitioners or researchers, are being implemented<br>- New resources as aids to learning have been co-designed and produced, with good feedback received from ACWs and community members<br>o One flip chart covers environmental health, one covers ARF prevention<br>- COVID impacted travel during 2020 and 2021
**Community awareness and empowerment**	- While participating ACWs and family members benefited from the project, there was limited evidence that the wider community benefited from stage 1 activities during Feb 1, 2018-Jan 31, 2019.	- After reviewing the formative evaluation findings, ACW's activities were extended beyond just families including someone with ARF or RHD, to everyone in the participating communities.
**Health provider education**	- ACWs did not have the capacity or authority to provide ARF/RHD education for health professionals.<br>- Having good working relationships with local services requires high time investment due to either high staff turnover, absences or other staffing issues	- Further work is needed on greater integration of project activities into the clinic including in-services, meetings with clinic medical and nursing staff, and communication with the RHD Control program
**Health and education service integration**	- A lack of formal arrangements between the research team and service providers meant ACWs were unable to sustain activity in this domain. This needs to be implemented by a well-staffed project team.	- Increased project team staffing is required to take on effective stakeholder engagement, increase involvement of schools in ARF prevention and detection and support streamlined pathways for

		children navigating health and education systems when managing a chronic condition

Table S5. Community-wide ARF and RHD occurrences during the study at Sites A, B and C.

		Baseline	Activity Phase Yr1	Activity Phase Yr2	Activity Phase Yr3
		February 1st, 2017 - Jan 31st, 2018	Feb 1st, 2018-Jan 31st, 2019	Feb 1st, 2019-Jan 31st, 2020	Feb 1st, 2020- Jan 31st, 2021
RHD first diagnosis		0	1	0	0
ARF ± RHD*	All	6†	7	1	3
	First diagnosis	6	5	1	0
	Recurrence	0	2	0	3

*when ARF is diagnosed, RHD may be concurrently diagnosed
†one was not enrolled.

Figure S3: Relationship between self-reported and clinic-diagnosed potential streptococcal infections per household

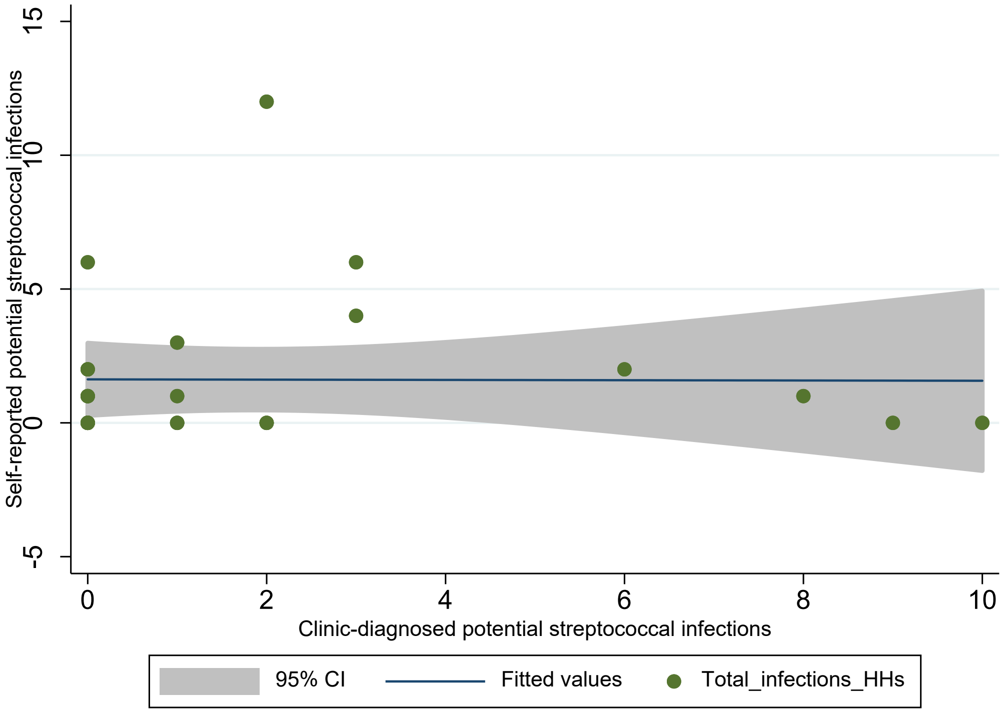

Figure S4: Schematic showing entry and exit from the study of the primary participants, new diagnoses of ARF or RHD, and preceding potential streptococcal infections

Yellow bars indicate period of secondary prophylaxis. Skin sores and sore throat occurrences (clinic data) are shown for those who had an ARF episode during the study.

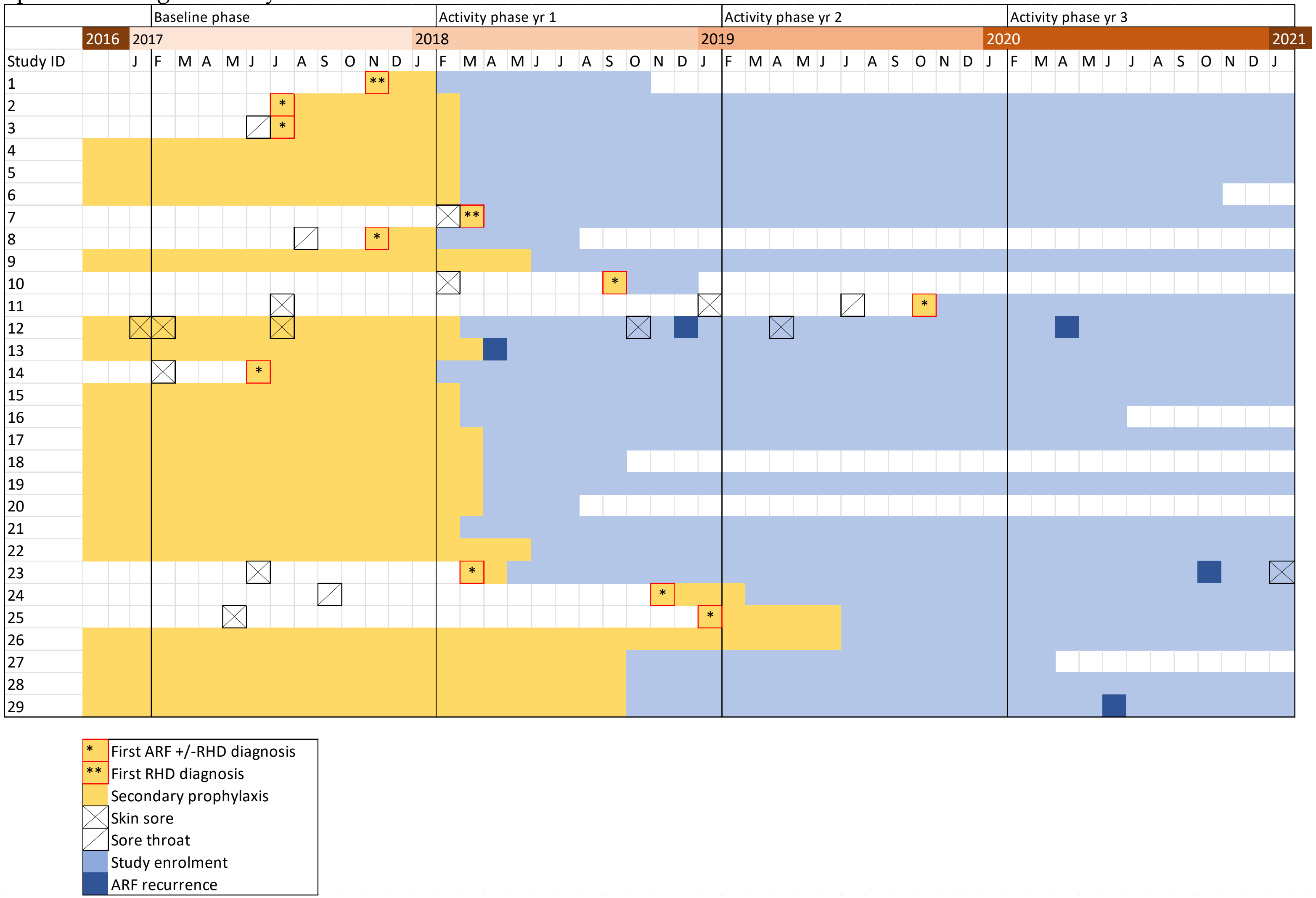

Table S6: Intramuscular benzathine benzylpenicillin dosing prior to recurrent ARF among primary study participants.

	Diagnosis	Phase of study when ARF recurrence occurred	Comment
Person 1	ARF recurrence POSSIBLE	Year 1	Had been getting monthly intramuscular benzathine benzylpenicillin doses but had had **10 consecutive days at risk** in the month prior to getting possible ARF recurrence
Person 2	ARF recurrence POSSIBLE	Year 1	Had intramuscular benzathine benzylpenicillin 23 days prior to the ARF recurrence diagnosis, but had had **17 consecutive days at risk** in the month prior. But ARF diagnosis in doubt, later revoked by a specialist then re-instated.
Person 2	ARF recurrence PROBABLE	Year 3	Had no recent intramuscular benzathine benzylpenicillin since secondary prophylaxis had been stopped by a specialist. Was then restarted.
Person 3	ARF recurrence DEFINITE	Year 3	Had intramuscular benzathine benzylpenicillin 10 days before the ARF recurrence diagnosis, but had had **42 consecutive days at risk** prior to that dose.
Person 4	ARF recurrence POSSIBLE	Year 3	Had intramuscular benzathine benzylpenicillin 16 days before the ARF recurrence diagnosis, but had had **23 consecutive days at risk** prior to that dose.

Figure S5: Status of health hardware reported from household surveys

Numbers indicate the number of surveys that reported on the item

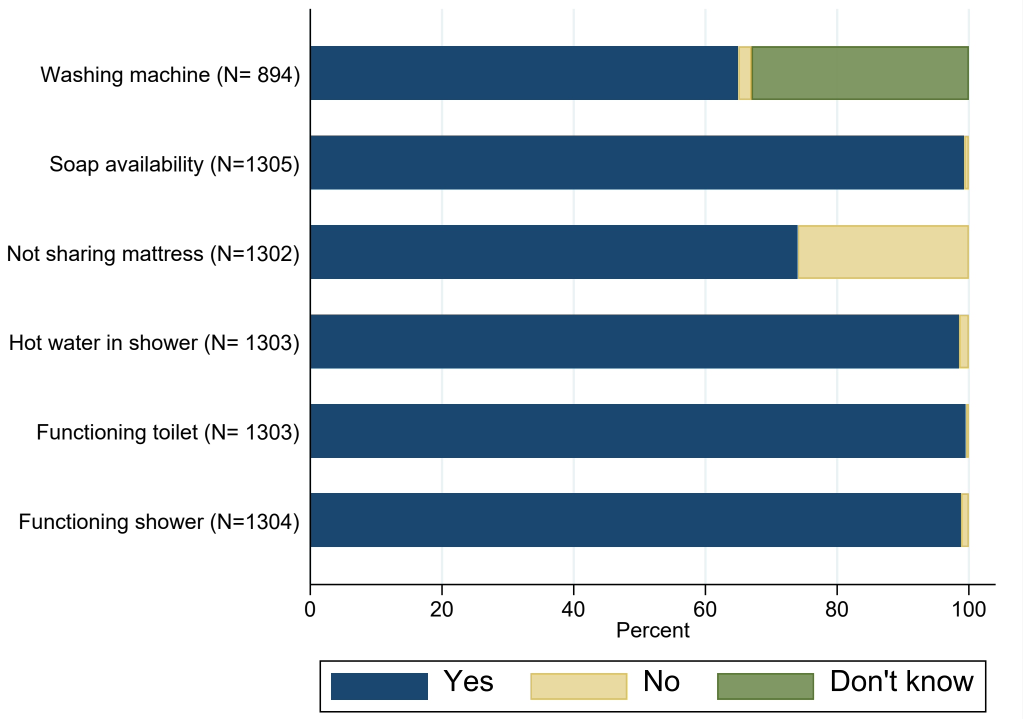

Supplement: Supplementary file 1 [file ijerph-19-10215-s001.zip › ijerph-1781826-supplementary.pdf]
